# Accelerative action of topical piperonylic acid on mice full thickness wound by modulating inflammation and collagen deposition

**DOI:** 10.1371/journal.pone.0259134

**Published:** 2021-10-26

**Authors:** Karina Gomes Moreira, Thais Paulino do Prado, Natália Ferreira Mendes, Renan de Medeiros Bezerra, Carlos Poblete Jara, Maria Helena Melo Lima, Eliana Pereira de Araujo

**Affiliations:** 1 School of Nursing, University of Campinas, Sao Paulo, Brazil; 2 Laboratory of Cell Signaling, Yokohama, Japan; 3 Obesity and Comorbidities Research Center, University of Campinas, Sao Paulo, Brazil; University of Messina, ITALY

## Abstract

Epidermal growth factor (EGF) promotes cell growth, proliferation, and survival in numerous tissues. Piperonylic acid, a metabolite present in peppers (*Piper nigrum* L. and *Piper longum* L.), can bind to the epidermal growth factor receptor (EGFR) and induce an intracellular signaling cascade leading to the transcription of genes responsible for these actions, especially in keratinocytes. These cells are fundamental in maintaining cutaneous homeostasis and are the first to be damaged in the case of a wound. Thus, we hypothesized that piperonylic acid improves wound healing. C57BL6/J male mice were submitted to dorsal skin wounds caused by a 6 mm punch and treated topically with piperonylic acid or vehicle. The wounds were evaluated macro- and microscopically, and tissue samples were collected for immunofluorescence and real-time PCR analyses on days 6, 9 and 19 post-injury. Topical piperonylic acid improved wound healing from day 6 post-injury until closure. This phenomenon apparently occurred through EGFR activation. In addition, piperonylic acid modulated the gene expression of interleukin (*Il*)-6, il-1β, tumor necrosis factor (*Tnf*)-α, il-10, monocyte chemoattractant protein (*Mcp*)-1 and insulin-like growth factor (*Igf*)-1, which are important for the healing process. By day 19 post-injury, the new tissue showed greater deposition of type I collagen and a morphology closer to intact skin, with more dermal papillae and hair follicles. We conclude that piperonylic acid may be a viable option for the treatment of skin wounds.

## Introduction

Pepper is one of the best-known spices around the world and has been used for medicinal purposes since ancient times [1]. Piperonylic acid (PA) is a metabolic derivative of the black pepper (*Piper nigrum* L.) and the long pepper (*Piper longum* L.) [1]. PA has several pharmacological actions, including antioxidant, antihypertensive, antiplatelet, antiasthmatic, analgesic, antitumor, antipyretic, antispasmodic, antidepressant, antidiarrheal, anxiolytic, anti-inflammatory, and immunomodulatory, among many others [1–4].

Wound healing is a public health problem worldwide and a huge challenge for health care professionals because it requires a lot of care time and a large amount of health resources [5,6]. It is a complex phenomenon that involves several cell types and proteins that communicate in a complex and intricate signaling network [7]. One of the important proteins involved in wound healing is epidermal growth factor (EGF) [8,9]. EGF stimulates cell growth, proliferation, and survival in various tissues [9]. Lee et al. [10] described that PA can activate the epidermal growth factor receptor (EGFR) and thus trigger an intracellular signaling cascade leading to the transcription of genes responsible for cell proliferation and survival, especially in cultured keratinocytes. Keratinocytes are fundamental cells in maintaining cutaneous homeostasis in the epidermal barrier and the first cells to be injured in the case of wounds [11,12]. The aim of this work was to evaluate the effect of topical treatment with PA on wound healing in an animal model.

## Methods

### Animals

Male C57BL6/J mice were received at 4 weeks of age from the Multidisciplinary Center for Biological Research (CEMIB/UNICAMP). They were kept in individual boxes, with standard rodent feed (Purina) and water provided *ad libitum*, and under a standardized light/dark cycle and temperature of 22 ± 2°C. After a week of adaptation, the animals were placed in individual cages and then, when they reached 8 weeks of age, they were randomly separated into two groups: the vehicle group, which received 0.1% dimethyl sulfoxide (DMSO) in Carbopol gel, and the PA group.

### Wound model

In the vehicle and PA groups, two wounds were made on the back of each animal. Before wounding, hair was removed with a razor and the skin was cleaned with 70% alcohol. General anesthesia was applied via intraperitoneal injection of 80 mg/kg ketamine hydrochloride and 8 mg/kg xylazine. The lesions were created in the dorsal region with a 6 mm metallic punch. The skin and subcutaneous tissue were removed until the muscular fascia was exposed. Immediately after the wound, the respective topical treatment was started. After the procedure, the animals received an analgesic—dipyrone (6 mg/kg)—via gavage and were placed in individual heated cages to avoid an abrupt drop in temperature.

Wounds were treated at the same time each day until the lesion was completely closed. The treatment was either 20 μL of 10 μM PA (purchased from Sigma-Aldrich) or the same volume of 0.1% DMSO in Carbopol gel. This concentration was based on data from Lee et al. [10].

### Evaluation of wound healing process

The wound healing process was evaluated using photographs acquired with a Sony Cyber Shot® digital camera (model DSC-S950S, 10 MP, 4× optical zoom) and an appropriate tripod to maintain an equal distance in all images. The photographs were taken daily by the same evaluator. When taking the photos, the animals were first placed in an acrylic box and anesthetized with isoflurane (BioChimico). The images were digitalized, and the wound area was measured using the Image J software (National Institutes of Health, Bethesda, MD, USA). The wound retraction is expressed as a percentage (%), measured by the following mathematical formula: (initial wound area)–(daily wound area) × 100 (home area).

Scar tissue was collected on days 6, 9, and 19 after injury from different groups of animals (five animals each). After collecting tissue, the animals were euthanized using a thiopental overdose and subsequent cervical dislocation. The tissue was stored at -80°C for future molecular and cellular evaluation.

During the experimental period, the mice were evaluated for motor activity, food acceptance, wound appearance (presence/absence of exudate), and death. Data were recorded for each mouse until the day of euthanasia, which was performed using a thiopental overdose and subsequent cervical dislocation.

### Histology

The dissected skin tissues were fixed by immersion in formaldehyde. They were processed as followed: immersed in increasing concentrations of alcohol (70%, 80%, 95%, and 100%), xylol, and paraffin; embedded in paraffin; sectioned at 5 μm; and adhered to a poly-l-lysine-coated microscopy slides. The sections were incubated for 30 s with hematoxylin, washed in distilled water, incubated for another 30 s with eosin, washed again in distilled water, and then dehydrated. The slides were mounted with Entellan® (Merck KGaA, Darmstadt, Germany) and then evaluated. The slides were examined with an optical transmission microscope (Zeiss, Oberkochen, Germany) with a 16X/2 objective lens and Zeiss Axiocam ICc5 camera. The images were processed using Image Pro-Plus software (Media Cybernetics, Rockville, USA).

### Immunofluorescence

Tissue samples were immersed in 4% formaldehyde overnight. Subsequently, they were washed three times with 1X phosphate-buffered saline (PBS), and then cryopreserved in 20% sucrose for 3 days and 40% sucrose for 1 week. Samples were then embedded in OCT (Merck KGaA, Darmstadt, Germany) and sectioned using a Leica CM1860 cryostat (Leica Biosystems, Leider Lane Buffalo Grove, USA). The sections were then subjected to immunofluorescence. Primary antibodies specific for keratinocyte markers were used: cytokeratin 10 (abcam, Cambridge, MA, USA, ab76318), cytokeratin 6 (abcam, ab93279), and EGFR (Santa Cruz, Santa Cruz Biotechnology, Texas, USA, sc-373746). The sections were incubated with primary antibodies, diluted 1/50, overnight, after which they were washed with PBS 7.4 and then incubated with secondary antibodies, diluted 1/100, for 2 hours (protected from light). The slides were assembled using vectashild® (Vector Laboratories, CA, USA) and coverslips. The analyzes were performed with confocal microscopy (Leica Microsystems, Teban Gardens Crescent, Singapore).

### Cell viability assay—Piperonylic Acid (PA) dilution

100mM PA stock solution was prepared with High Glucose DMEM (serum free) and Piperonylic Acid (Sigma, cat: P49805 powder). First, DMEM (Thermo Fisher Scientific, CA, USA) was heated at 60–80°C using a standard microwave (Panasonic NN-ST45KWBPQ) before adding the Piperonylic Acid. Next, the 100mM PA stock solution was mechanically homogenized for 15 minutes (1500 rpm). Then, the pH was adjusted to 7.5 using NaOH 5N. The heating and homogenization procedures were repeated until not macroscopically crystals were visible in the solution at 7.5 pH. The pH of the DMEM solution (Control) was adjusted when necessary. 100mM PA stock solution and control treatments were filtered in a sterile environment using 0.22um filter-syringe system. Fresh 10mM, 1mM and 100uM solutions were obtained by serial dilution using the 100mM PA stock solution with DMEM, and then applying to the HaCaT cell.

HaCaT cells (5 × 104) were seeded in DMEM supplemented with 10% fetal bovine serum (FBS) and 1% penicillin-streptomycin in 96-well plates and incubated for 24 h. Then, the cells were washed with PBS three times and treated with 10mM, 1mM and 100uM concentrations that were obtained by serial dilution from the 100mM solution in pH at 7.5. After 24 or 48 h, the cells were treated with thiazolyl bromide blue tetrazolium (MTT; prepared as 0.45 mg per mL) and incubated for 1.5 h. The formazan crystals were dissolved in 100 μL DMSO and the plate was read at 570 nm in GloMax® Discover Microplate Reader (Promega Corporation Biotechnology, WI, USA).

### RNA extraction

The tissue samples were mixed in TRIzol reagent (Invitrogen, São Paulo, Brazil) for 30 s using specific equipment for this purpose (Polytron-Aggregate, Kinematica, Littau/Luzern, Switzerland) at maximum speed. Subsequently, the homogenates were centrifuged at 6,000 *g*, and total RNA was isolated according to the manufacturer’s instructions and quantified by spectrophotometry. RNA integrity was assessed by agarose gel electrophoresis. Complementary DNA (cDNA) was synthesized from 2 μg total RNA using the High-Capacity cDNA Reverse Transcription Kit (Applied Biosystems, CA, USA).

### Real-time PCR

Five nanograms of RNA were subjected to reverse transcription using random hexamer primers and Superscript Maloney MLV reverse transcriptase (Promega Corporation Biotechnology, WI, USA). The cDNA integrity was evaluated by performing electrophoresis on 3% agarose gel. Real-time PCR reactions were performed using the TaqMan system (Applied Biosystems), which consists of a pair of primers and a probe marked with a fluorophore. GAPDH was used as the endogenous control. The gene expression was determined by analyzing the results in the 7500 System SDS Software program (Applied Biosystems).

### Statistical analysis

The results are presented as mean ± standard deviation. For statistical analysis, we applied the independent samples Student’s t-test (for two groups) or analysis of variance (ANOVA) (for more than two groups). When necessary, Tukey’s test was used to determine differences between groups. The level of significance for rejecting the null hypothesis was 5% (p < 0.05).

### Ethical aspects

The ethical principles for the use of animals established by the Federal Constitution of Brazil were followed. This study was approved by the Ethics Committee for Animal Use in Research of the Institute of Biology of the University of Campinas–UNICAMP—CEUA # 5414-1/2019.

## Results

### PA improved wound healing in mice and was not toxic to HaCaT cells

**Fig 1:**
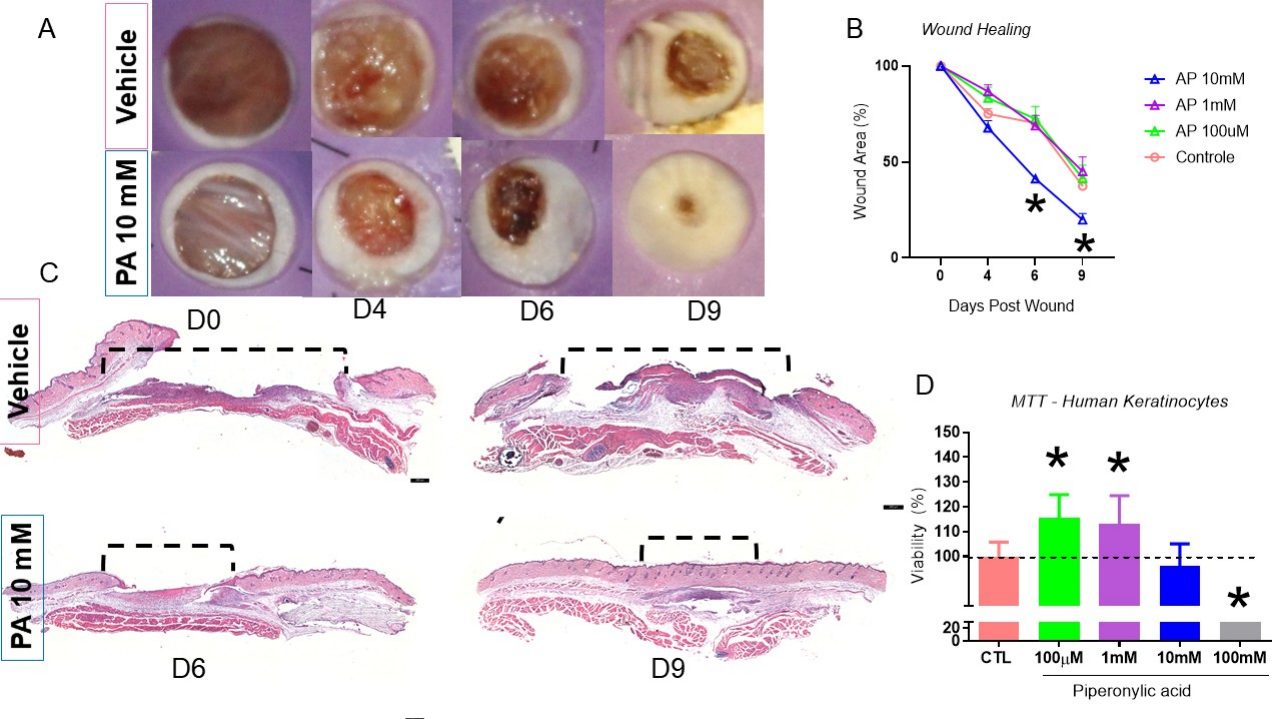
The macroscopic and microscopic aspects of the wounds treated for nine days with topical PA versus vehicle and HaCaT Cells treated 24 hours with PA in different concentrations for Viability Assay (MTT). Wounds were photographed (A) (representative of five distinct experiments). Wounds were treated with PA in100μM, 1mM, 10mM, 100mM were measured during the healing process from day 0 to day 9 (B) n = 5; *p<0.05 and ***p<0.01 vs. vehicle. Microphotograph of hematoxilin/eosin staining of the wounds treated for nine days with 10mM PA vs. vehicle (C) n = 5. MTT assay, HaCaT cells incubated for 24 hours with in100μM, 1mM, 10mM, 100mM vs. vehicle (D) n = 3, *p<0.05.

We first carried out a dose-response experiment to determine which dose of PA to use, based on the concentration of 100 μM used in a previous study in cultured keratinocytes [10]. For this, we used 20 C57BL/6 mice randomly divided into four groups: vehicle, 10 mM PA, 1 mM PA, and 100 μM PA. Each mouse was treated daily until day 9 post-injury. We found that the 10 mM PA group showed a significantly improved healing time from day 6 post-injury (Fig 1A and 1B). Therefore, we evaluated reepithelization on days 6 and 9 post-injury. We found that 10 mM PA led to an earlier healing process; the wound showed a smaller area of non-epithelized tissue (Fig 1C).

PA is an acidic substance, and thus we evaluated whether it reduced cell viability using the MTT assay. The immortalized human keratinocyte cell line HaCaT was divided into vehicle, 100 μM PA, 1 mM PA, 10 mM PA, and 100 mM PA groups. PA was not cytotoxic at 100 μM, 1 mM, and 10 mM concentrations (Fig 1D).

**Fig 2:**
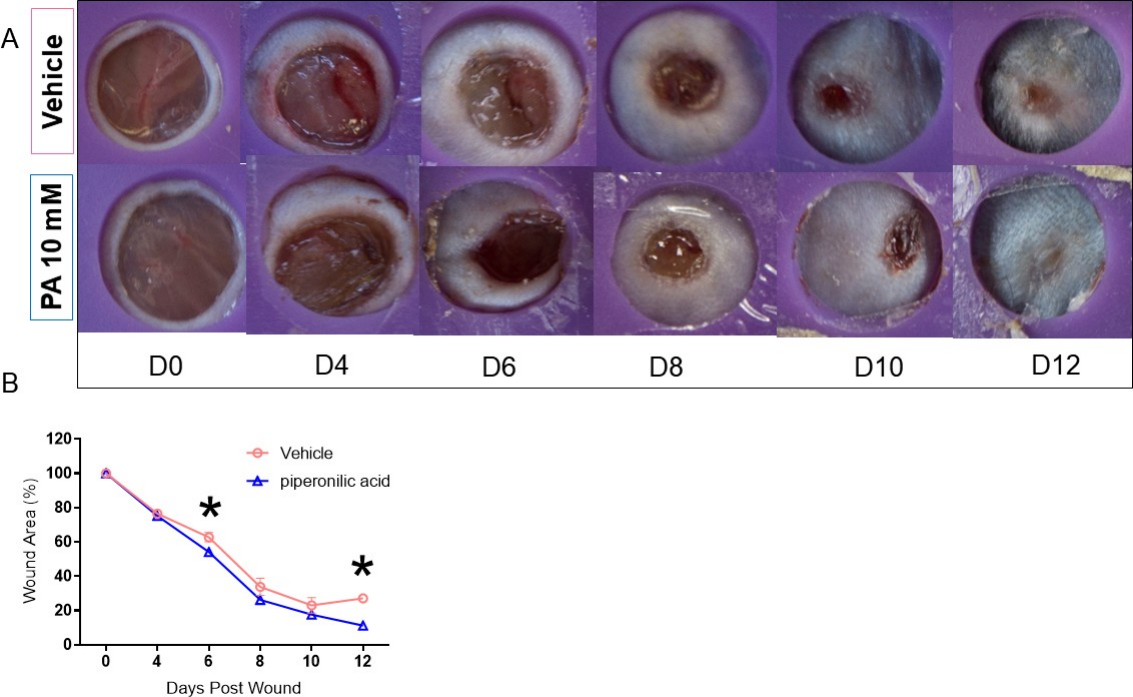
The macroscopic aspects of the PA 10mM vs. vehicle treated wounds until day 12. Wounds were photographed (A) (representative of five distinct experiments). Wounds were treated with 10mM PA vs. vehicle were measured from day 0 to day 9 (B) n = 5 *p<0.05.

Based on the above results, we conducted the subsequent experiments using 10 mM PA. We next followed the healing process until the lesion was completely closed, with two groups (n = 5 per group): vehicle and 10 mM PA. We verified once again that there was a significant improvement in wound closure in the PA group on days 6 and 12 post-injury, at which time the PA group was completely healed (Fig 2A and 2B).

**Fig 3:**
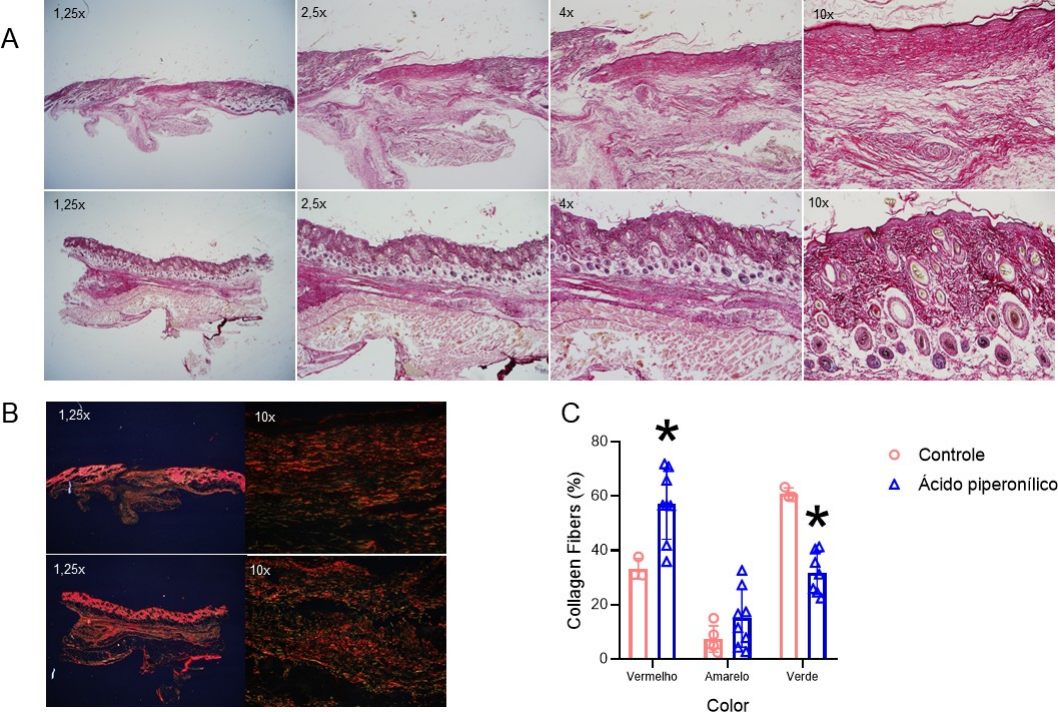
Microscopic aspects of the wounds. Microphotograph of hematoxilin/eosin staining of 5.0 μm sections of wounds on day 19. Arrows showing vessels, hair follicle and papillae structure (A). Collagen deposition stained by picrosius red on day 19 (B). Determination of type I and type III collagen fibers by optical density of red, yellow, and green values (C). *p<0.05. The images are representative of five distinct experiments.

To determine whether PA leads to the formation of better quality reepithelialized tissue, we evaluated the scar tissue morphology on day 19 post-injury. For this, we used 10 animals randomly divided into the two groups (vehicle and 10 mM PA). Based on hematoxylin and eosin staining, most wounds were completely reepithelialized when assessed microscopically regardless of the treatment group. However, PA treatment led to tissue with better morphological characteristics, with more structures such as hair follicles, blood vessels, and dermal papillae, suggesting new reepithelization with greater adhesion between the epidermis and dermis (Fig 3A). In addition, there were differences in the deposition of type I (red) and type III (green) collagen fiber between the two groups, especially greater deposition of type I collagen in the PA group. Moreover, these fibers were thicker and more interlaced, denoting newly formed, better-quality tissue that was more similar to uninjured tissue (Fig 3B).

### PA modulated the expression of genes involved in the healing process

**Fig 4:**
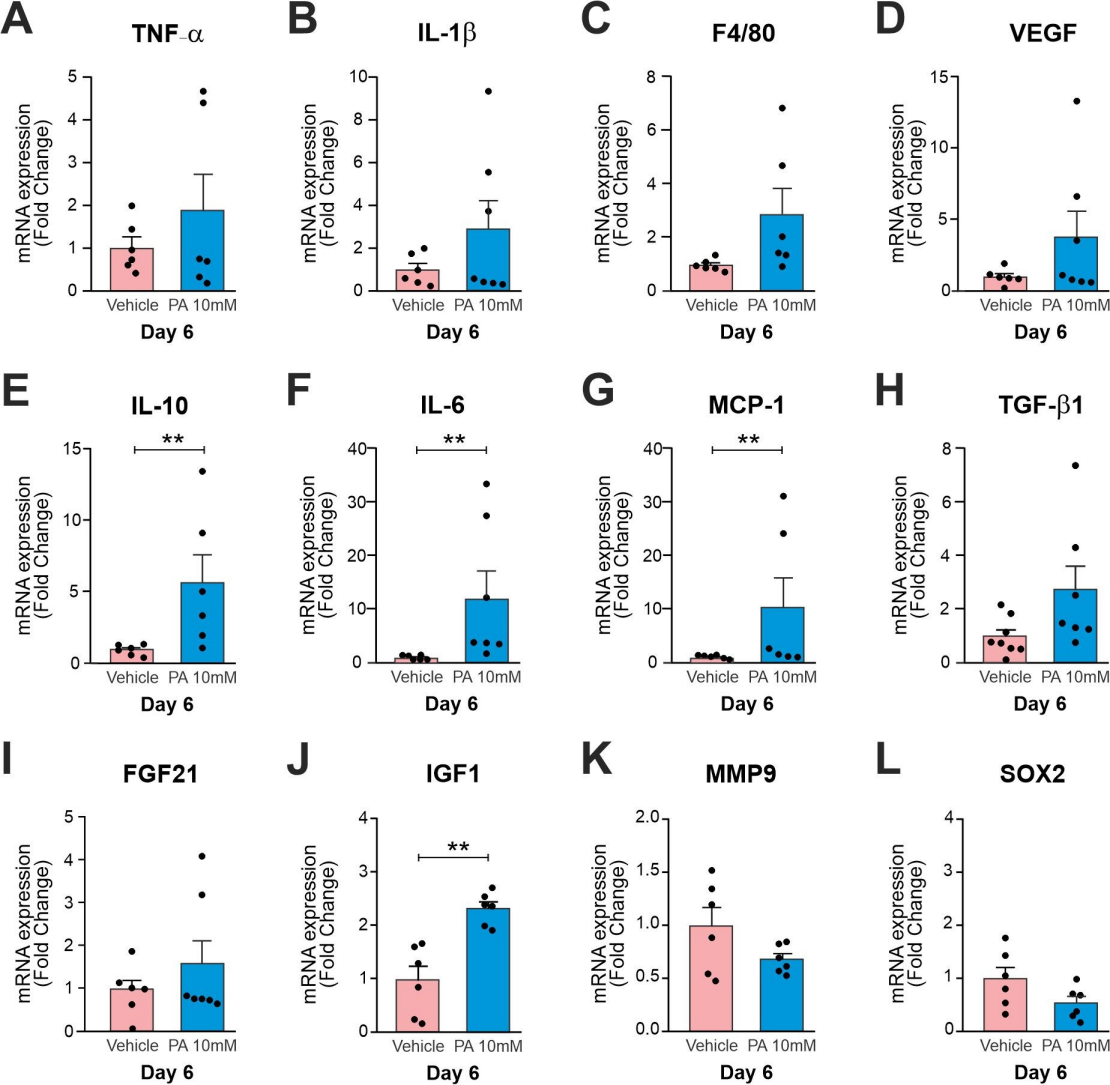
Expression of transcripts in the wounds on day 6 treated with 10 mM PA vs. vehicle. Real time PCR were employed to determine the expression of transcripts encoding for *Tnf-α* (A), *Il-1β* (B), *F4/80* (C), *Vegf* (D), *Il-10* (E), *Il-6* (F), *Mcp-1* (G), *Tgf-β1* (H), *Fgf21* (I), *Igf-1* (J), *Mmp-9* (K), and *Sox2* (L). In all experiments n = 6 *p<0.05.

We next investigated changes in the expression of genes related to the healing process at days 6 and 9 post-injury. Initially, we used 12 animals randomly divided into the two groups (vehicle and 10 mM PA) euthanized on day 6 post-injury. *Tnf-α*, *Il-1β*, module-containing mucin-like hormone receptor-like 1 also known as *f4/80*, vascular endothelial growth factor (*Vegf*), il-10, interleucina-6 (*Il-6*), monocyte chemoattractant protein-1 (*Mcp-1*), transforming growth factor beta 1 (*Tgf-β1*), fibroblast growth factor 21 (*Fgf21*), insulin-like growth factor 1 (*Igf-1*), matrix metallopeptidase 9 (*Mmp-9*) and sry-box transcription factor 2 also known as *Sox2* were evaluated (Fig 4A–4L). There was a significant increase in *Il-10*, *Il-6*, *Mcp-1* and *Igf-1* (Fig 4E–4G and 4J).

**Fig 5:**
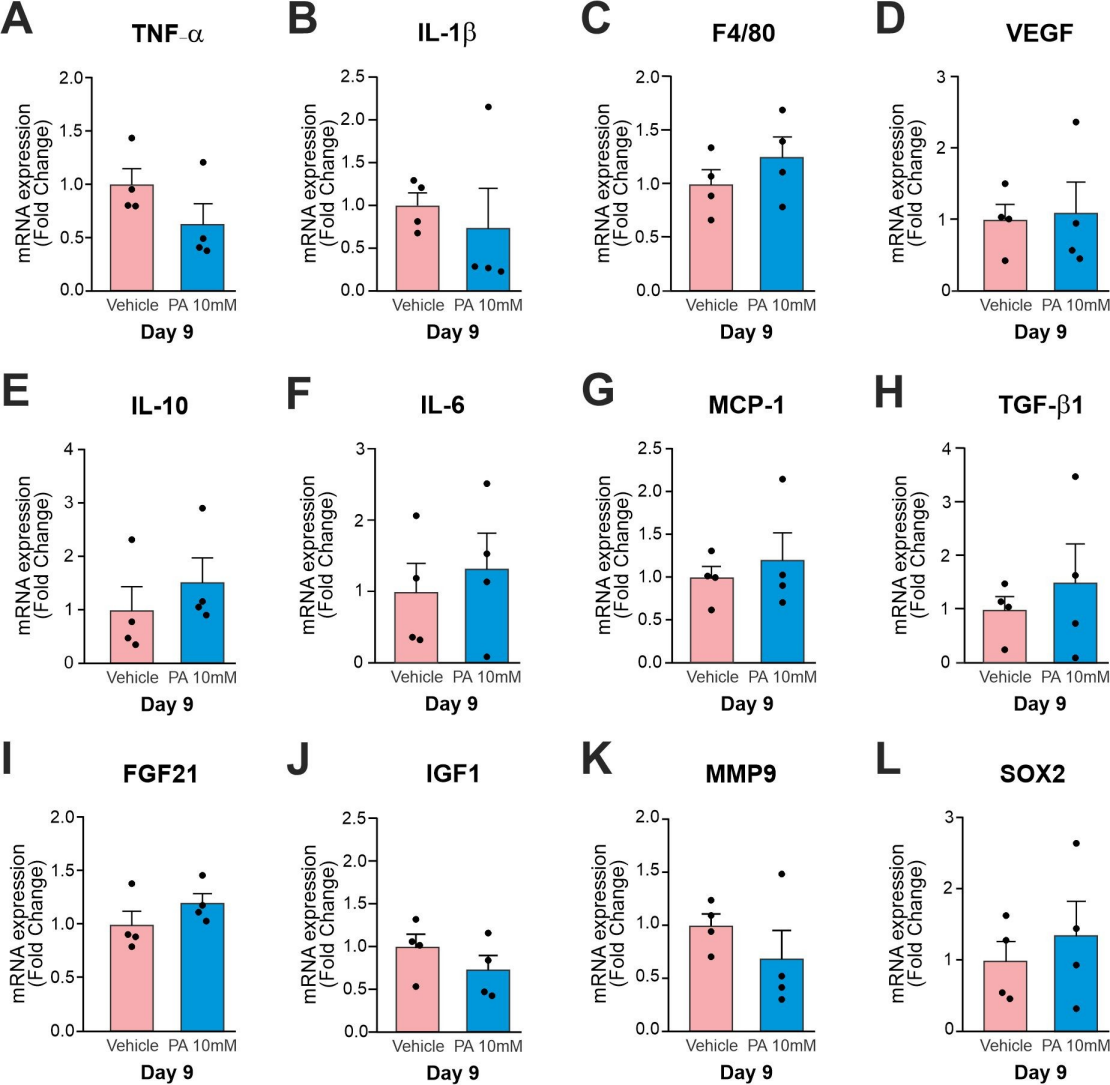
Expression of transcripts in the wounds on day 9 treated with 10 mM PA vs. vehicle. Real time PCR were employed to determine the expression of transcripts encoding for (A), *Il-1β* (B), *F4/80* (C), *Vegf* (D), *Il-10* (E), *Il-6* (F), *Mcp-1* (G), *Tgf-β1* (H), *Fgf21* (I), *Igf-1* (J), *Mmp-9* (K), and *Sox2* (L). In all experiments n = 4.

On day 9 post-injury, we used 10 animals that were randomly divided into two groups (vehicle and 10 mM PA). We did not observe a significant modulation in genes expression in this day (Fig 5A–5L).

### PA positively modulated EGFR expression

**Fig 6:**
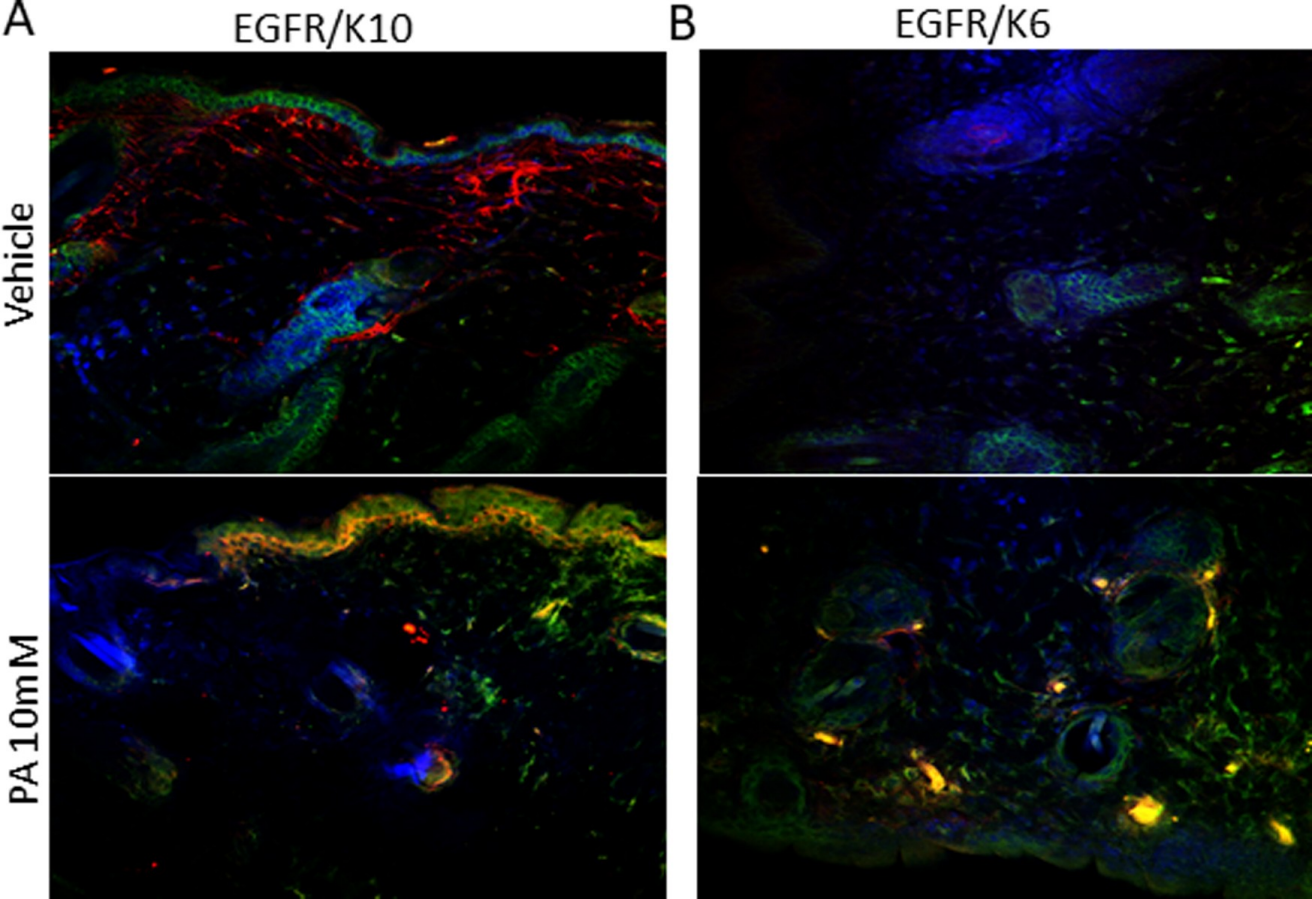
Immunofluorescence of the wounds on day nine treated with 10 mM PA vs. vehicle. Cytokeratin 10 staining for keratinocytes (green) and EGFR (red) (A). Cytokeratin 6 staining for keratinocytes (green), EGFR (red) and the merge (yellow) (B). DAPI labeling nuclei (blue). Representative of three distinct experiments.

We evaluated whether PA treatment modulated EGFR expression in epidermal cells using immunofluorescence. PA seemed to modulate positively the expression of EGFR in keratinized stratified epithelial cells and proliferative squamous epithelial cells, labeled with anti-cytokeratin 10 and 6, respectively, and colocalized with EGFR (Fig 6).

## Discussion

We demonstrated that PA improved wound healing, providing a better-quality scar with characteristics closer to uninjured skin. Previous data in the literature [10] and the results of this study suggest that these events occur through the activation of EGFR, which is a component of the EGF family and is predominantly distributed in keratinocytes [9]. EGFR knockout mice present a serious impairment of the healing process, with delayed wound closure accompanied by a decrease in keratinocyte migration and increased edema [13,14]. EGF regulates multiple aspects of cutaneous wound healing, including inflammation, wound contraction, proliferation, migration, and angiogenesis [15,16]. EGFR is expressed throughout the epidermis, with greater importance in the basal layer [17]. In this study, we demonstrated that EGFR is expressed in stratified and proliferative squamous epithelial cells, marked by cytokeratin 10 and 6, respectively. PA treatment seemed to increase EGFR expression in the stratified epithelium and in the hair follicle bulb (Fig 6).

Lee et al. (10) demonstrated in cultured keratinocytes that PA activates EGFR and promotes a series of intracellular actions such as positive modulation of *Egr1*, *C-Fos*, *C-Jun*, and *C-Myc* gene expression, all of which are involved in cell growth and survival. Those authors also demonstrated that PA acts by modulating the phosphoinositide 3-kinase/AKT and mitogen-activated protein kinase/extracellular signal-regulated kinase signaling pathways, which are responsible for activating genes involved in growth and survival [10]. In this study, we demonstrated that 10 mM PA applied topically to wounds on mice improved wound healing as early as day 6 post-injury; this healing was maintained until day 12 (Figs 1A–1C and 2A and 2B).

Furthermore, we demonstrated in HaCaT cells that PA was not cytotoxic at concentrations of 100 μM, 1 mM and 10 mM, and there was an increase in cell proliferation at 100 μM and 1 mM, which corroborates with the data describing activation in genes involved in cell proliferation signaling (Fig 1D). Nevertheless, we considered that the increase in cell proliferation could be harmful in late stages of wound healing potentially leading to hypertrophic scars [18]. Thus, wound healing was followed up until day 19 after injury and, as shown by H/E, we demonstrated an improvement in morphological characteristics of the scar (Fig 3A).

The skin is a complex organ composed of the epidermis, dermis, and appendages, including hair follicles and sebaceous glands. Wound healing in adult humans results in scarring without any cutaneous appendages [19]. Several of the molecular and cellular events that orchestrate wound healing in mammals have been elucidated; however, therapeutic options that lead to perfect regeneration of scarless skin, including the formation of appendages in adult skin, are still lacking [19]. Previous studies have shown that mice under normal conditions have new hair follicles in the wound area, due to the development of the embryonic follicle [20,21]. Our study revealed that topical PA led to the formation of an epithelium more like intact skin, with the presence of more structures like hair follicles. In addition, there appeared to be greater formation of dermal papillae (Fig 3A). These structures provide better exchange of nutrients, oxygen, and waste products between the dermis and epidermis and increase the surface contact between these skin layers, preventing their separation. Dermal papillae also play a key role in the formation, growth, and cycle of hair follicles [22]. Dermal papillae are responsible for holding active fibroblasts and efficiently synthesizing and depositing all components of the extracellular matrix, such as glycoproteins, proteoglycans, and elastic fibers [22]. Thus, we believe that PA may have a synergistic effect by improving the formation of dermal papillae, which in turn may have influenced the development more hair follicles in the neo-formed area.

We also showed by picrosirius staining that PA increased the deposition of type I collagen fibers (Fig 3B). In uninjured adult skin, the ratio of collagen I to collagen III is approximately 4 to 1. In the dermis formed after the wound, collagen type III is temporarily increased and is slowly degraded during the remodeling phase [23,24]. During wound healing, the collagen matrix supports and controls fibroblast migration and activity; it acts as sustenance and participates in signaling related to angiogenesis, granulation tissue, and reepithelization [23,24]. Collagen III is quickly deposited along the initial matrix, acting as a barrier to pathogens and fluid loss. In the advanced stages, type III collagen is degraded by proteases and replaced by type I collagen, which takes longer to deposit but presents greater physical resistance [23,24]. In our study, PA may have led to increased collagen deposition in the proliferative and remodeling phase, which may have also influenced the faster lesion closure (Fig 3B and 3C).

PA, the main extract of *P*. *longum* and *P*. *nigrum*, has an extensive history of medicinal use and exhibits a variety of biochemical and pharmaceutical properties, including anti-inflammatory, antioxidant, and antimicrobial activities [1–4,25]. Thus, its use should lead to anti-inflammatory effects during wound healing. IL-10 is known as a key mediator of the pro- to anti-inflammatory transition and in the control of scar formation [26]. IL-10 overexpression accelerates reepithelialization, the formation of granulation tissue, and neo-angiogenesis, in addition to reducing the size of the scar [26–29]. In our study, IL-10 was significantly increased on days 6 post-injury, change that may also have contributed to the early wound closure (Fig 4E).

In recent years, the different phenotypes of macrophages, namely M1 and ​​M2, have been widely studied in numerous areas including wound healing. M1 macrophages phagocytize undesirable products and have numerous functions in tissue repair when they polarize to the M2 phenotype [30]. Studies have shown that this polarization is characterized by the increased release of pro-inflammatory cytokines (TNF-α, IL-1β, IL-6), chemokines (MCP-1) and growth factors such as IGF-1, VEGF-α, TGF-β among other changes [31,32].

In our study, the expressions of *Il-10*, *Il-6*, *Mcp-1* and *Igf-1* genes were increased (Fig 4E–4G and 4J). These changes suggest that M1 macrophages shifted to the M2 phenotype reestablishing tissue homeostasis as anti-inflammatory cells, and enhancing macrophage-derived matrix metalloproteinases that are involved in extracellular matrix digestion and collagen renewal [31,33]. Moreover, macrophages have been identified as the main source of myofibroblasts in a process known as the macrophage-myofibroblast transition (MMT) [34]. During the proliferation stage, macrophages actively signal to dermal fibroblasts. A subset of CD206+/CD301b+ macrophages induce the transition from fibroblasts to myofibroblasts in mice and humans, increasing the deposition of collagen and α-smooth muscle actin (α-SMA) in the wound [35].

## Conclusion

PA is a readily available and low-cost product that could be a viable therapeutic option to improve wound healing by modulating important genes involved in this process without having any apparent unwanted effects. New studies need to be carried out to identify in which types of wounds PA would be more indicated, as a chronic wounds and to investigate more deeply the interactions between PA and EGFR.
